# Neonatal brain MRI to prognosticate neurodevelopmental outcomes in fetal growth restricted infants: a systematic review

**DOI:** 10.3389/fped.2025.1681205

**Published:** 2026-01-12

**Authors:** Randy Ramdial, Madeline A. MacNamara, Randal Moldrich, Paul B. Colditz, Julie A. Wixey

**Affiliations:** 1 UQ Centre for Clinical Research, Faculty of Health, Medicine and Behavioural Sciences, The University of Queensland, Brisbane, QLD, Australia; 2 Department of Neurology, Perth Children’s Hospital, Perth, WA, Australia; 3 Perinatal Research Centre, Royal Brisbane and Women’s Hospital, Brisbane, QLD, Australia

**Keywords:** brain, fetal growth retardation, intrauterine growth restriction, magnetic resonance imaging, neurodevelopment, newborn

## Abstract

**Aim:**

The purpose of this study was to identify magnetic resonance imaging (MRI) brain markers at birth that prognosticate neurodevelopmental outcomes at ≥12 months of age in fetal growth restricted (FGR) infants.

**Methods:**

A systematic literature search was undertaken in PubMed, Embase, Cumulative Index to Nursing and Allied Health Literature (CINAHL), and Scopus in March 2025. Articles were assessed by two reviewers. The inclusion criteria included papers relating to infants with birth weight <10th percentile, with assessed neurodevelopmental outcomes at 12 months of age or older, and MRI performed at birth with markers for neurodevelopment, and comparison of data to appropriate for gestational age (AGA) infants. The quality of studies was assessed using the Cochrane-approved Quality in Prognosis Studies tool.

**Results:**

Three articles met the inclusion criteria. All demonstrated a correlation between MRI in regions of the brain and neurodevelopmental outcomes >12 months of age across all studied infants. However, only one of the three studies correlated early MRI results with neurodevelopmental outcomes specifically in FGR infants and thus a meta-analysis could not be performed. The single study reports a positive correlation between MRI total parenchyma area and cognitive scores in FGR infants.

**Conclusion:**

The current literature highlights the developmental risk in FGR vs. AGA infants. FGR neonates have significantly different MRI results compared to AGA neonates and MRI findings in the neonate are associated with adverse neurodevelopmental outcomes. However, current evidence is insufficient to firmly establish MRI prognostic capabilities specifically for FGR infants. Sub-group analysis of the FGR cohort in the reported studies and the use of more advanced MRI techniques would likely elucidate this. Further research is required to ascertain robust clinical MRI markers of early adverse brain development in the FGR newborn.

**Systematic Review Registration:**

identifier CRD42023400436

## 1 Introduction

Fetal growth restriction (FGR) results in a fetus not reaching its anticipated biological growth and is most commonly due to insufficient placental function (1). FGR is estimated to occur in 3%–7% of all pregnancies worldwide with an increased incidence in low and lower-middle income countries (2). Growth restricted fetuses have an increased risk of short and long-term health complications that may impact quality of life. These infants are at an increased risk of poor neurodevelopmental outcomes including learning, motor, cognitive and behavioural difficulties (3–7) with neurodevelopmental disorders reported in 24%–53% of FGR infants (8, 9). Therefore, it would be advantageous to be able to predict those FGR neonates at birth who are likely to have an adverse outcome. Early detection of infants at risk is critical for early and effective intervention to reduce neurodevelopmental burdens (10, 11).

Magnetic resonance imaging (MRI) is a non-invasive method used to assess brain structure and can be used to assess functional networks and microstructure. Neuroimaging studies using MRI have been investigated as potential methods of screening for neonatal brain injury. Neonatal MRI is relatively sensitive and has the ability to detect subtle neuropathology by high-resolution visualisation of structural changes (12). Previous studies have documented MRI brain correlations with neurodevelopmental outcomes in preterm infants (13–15). In infants with FGR, MRI studies have shown structural brain changes at birth that persist at 1 year of age (12, 16). Although changes in brain morphology are observed more frequently in FGR infants, not all morphological differences correlate with neurodevelopmental outcome. Early detection and prognostication are crucial to mitigating poor neurodevelopmental outcomes (10, 11). Multiple studies examine the link between brain morphology using MRI and neurodevelopmental outcomes in FGR infants (17–19). We hypothesize that MRI markers at term can prognosticate neurodevelopmental outcomes in FGR infants ≥12 months of age. This study aims to perform a systematic review of the existing literature to identify MRI markers that may prognosticate neurodevelopmental outcomes in FGR infants.

## 2 Methods

This review was registered with the International Prospective Register of Systematic Reviews (PROSPERO) July 2023 (ID: CRD42023400436) and designed in accordance with the PRISMA guidelines (20).

### 2.1 Search

A comprehensive search of the literature was undertaken in PubMed, Embase, Cumulative Index to Nursing and Allied Health Literature (CINAHL), and Scopus in March 2025 for relevant papers in English and limited to humans, with similar search strategies. For PubMed, the search was: (“small for gestational age”[tiab] OR “fetal growth restriction”[tiab] OR “foetal growth restriction”[tiab] OR “intrauterine growth restriction”[tiab] OR “Growth restricted”[tiab] OR “Infant, Low Birth Weight”[Mesh]) AND “Magnetic Resonance Imaging”[Mesh] AND (“development”[tiab] OR “growth”[tiab] OR “growth AND development”[tiab] OR “cognition”[tiab] OR “cognitive development”[tiab] OR “Neurodevelopmental outcomes”[tiab] OR “developmental delay”[tiab] OR “cognitive impairment”[tiab] OR “motor impairment”[tiab] OR “language delay”[tiab] OR “behavioral outcomes”[tiab] OR “Bayley*”[tiab] OR “Griffith*”[tiab]).

### 2.2 Eligibility criteria

The inclusion criteria were: (1) primary peer reviewed publication, (2) relating to infants at less than the 10th percentile of birth weight, with assessed neurodevelopmental outcomes at 12 months of age or older, (3) relating to MRI markers for neurodevelopment at birth, (4) comparison of data to appropriate for gestational age (AGA) infants with assessed neurodevelopmental outcomes at 12 months of age or older and no signs of FGR. All inclusion criteria were to be met. Studies were excluded if they: (1) were reported in a non-English language, (2) were conducted in paediatric populations with neurological abnormalities and neonatal medical complications, (3) were commentaries, abstracts, or single case reports.

### 2.3 Study selection

After removal of duplicates using EndNote and Covidence, two authors R.R. and M.A.M. independently screened titles and abstracts to create a list of articles for a full-text review. Conflicting viewpoints were discussed until consensus was reached or resolved by J.A.W. The full-text review was then completed with respect to meeting the inclusion and exclusion criteria.

### 2.4 Risk of bias in individual and across studies

The quality of the included studies was assessed with the Cochrane- approved Quality in Prognosis Studies tool (21). All domains were scored as low, moderate, or high risk, and each consisted of multiple elements. A domain was considered at low risk of bias if most items were properly covered.

### 2.5 Summary measures

The principal summary measure was neurodevelopmental outcome at a minimum age of 12 months correlated with neonatal MRI findings. Data were often inconsistently presented between each study making it difficult to synthesise the outcomes, including data values, and particularly for FGR v AGA comparisons, therefore a meta-analysis could not be performed. A review of the findings is therefore presented.

## 3 Results

### 3.1 Study selection

**Figure 1:**
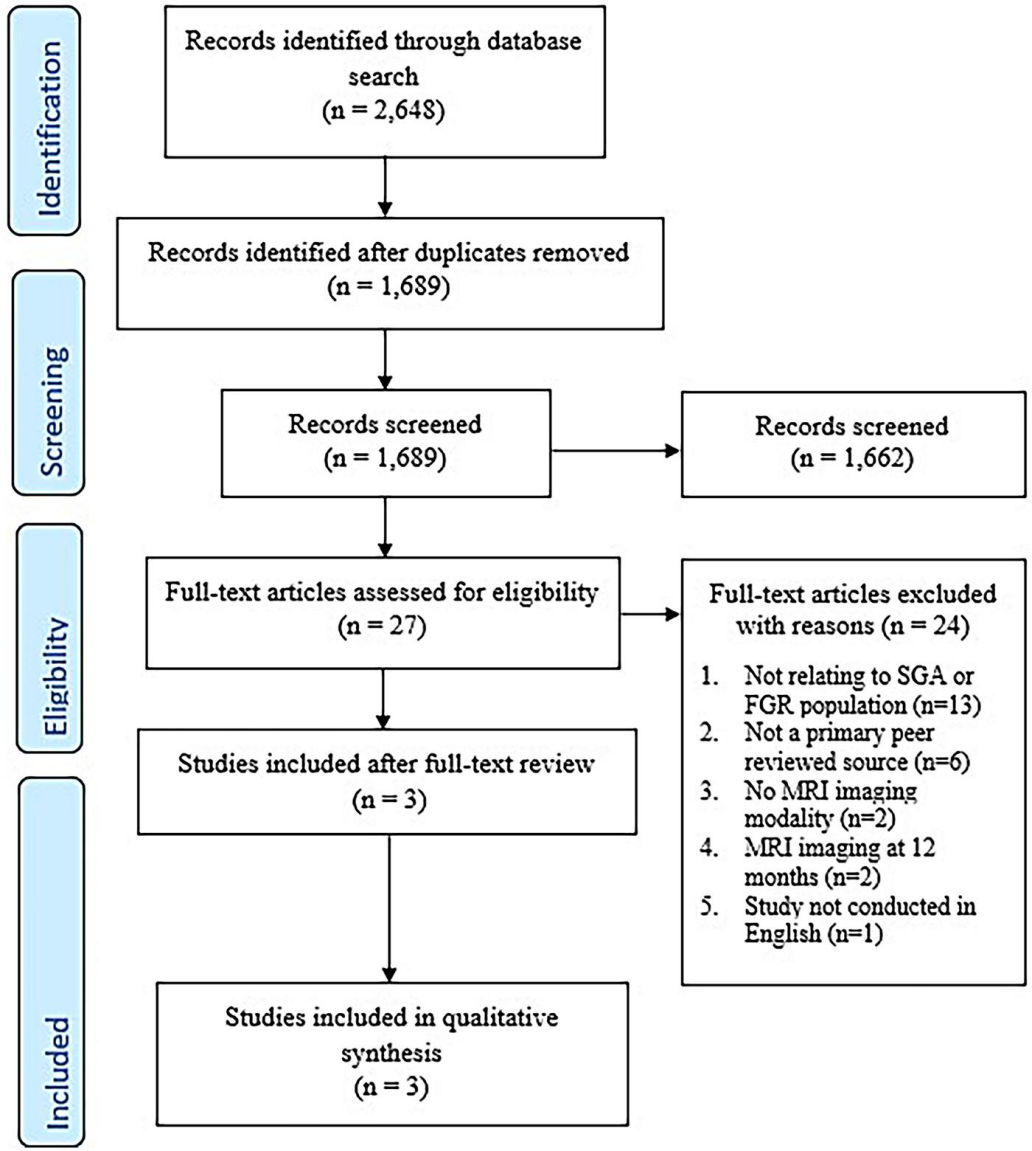
Study selection flowchart.

Excluding duplicates, 1,689 records were retrieved across the four databases searched (Figure 1). 27 studies remained for full-text review after the initial title and abstract screening. 24 of these articles were excluded as they did not relate to FGR population (*n* = 13), not a primary peer reviewed source (*n* = 6), no MRI imaging (*n* = 2), MRI imaging at 12 months (*n* = 2) and study not conducted in English (*n* = 1) (Supplementary Table S1). Disputes were resolved by J.A.W for 5 full-text reviews. This resulted in three studies included for analysis.

### 3.2 Quality of included studies

Table 1 represents assessment of risk of bias in individual studies. One study demonstrated moderate/high quality, one moderate, and one low/moderate.

**Table 1:** Assessment of risk of bias in individual studies.

Study	Study participation	Study attrition	Prognostic factor	Outcome	Study confounds	Statistical analysis	Total quality based on risk of bias
Brembilla et al. (19)	+	?	−	−	?	−/?	Moderate
Lodygensky et al. (17)	?	+	−	−	+	?	Low/Moderate
Sacchi et al. (18)	−	−	?	−	?	−	Moderate/High

High (+), low (−), and moderate (?) risk of bias.

### 3.3 Patient characteristics

Table 2 shows the patient characteristics of the three included studies, published between 2008 and 2021. Mean gestational age in included studies ranged between 29.4 and 32.1 weeks and mean birth weight ranged between 897 and 1,140 g. Mean corrected age at MRI ranged between 39.7 and 42.4 weeks and mean age at follow up ranged between 22 and 24.4 months. Included articles were from European countries, Italy, Switzerland, and England.

**Table 2:** Patient characteristics.

Study	GA, week, mean (SD)	BW, g, mean (SD)	Corrected age at MRI, week (SD)	Age at follow-up, months (SD)
Brembilla et al. (19)	29.4 ± 1.4	897 ± 214 g	39.7 ± 1.1	24.4 (IQR = 14.6–26.8)
Lodygensky et al. (17)	32.1 ± 2.5	1,140 ± 265	40.2 ± 0.7	24 (corrected age)
Sacchi et al. (18)	30	1,025	42.4	22 (IQR = 0.98)

SD, standard deviation; GA, gestational age; BW, birth weight; g, grams; MRI, magnetic resonance imaging.

### 3.4 Imaging characteristics

Table 3 shows MRI data acquisition and analysis of the three articles. Sacchi et al. and Lodygensky et al. employed high-resolution MRI with sub-2 mm slice thickness and automated post-processing protocols. Sacchi used PCA-based volumetry with a gestational age-specific atlas, while Lodygensky applied voxel-based morphometry (VBM) and k-nearest neighbour (k-NN) tissue classification, alongside manual segmentation of the hippocampus. Brembilla et al., by contrast, used lower-resolution 3 mm slices and manual 2D measurements of brain structures in combination with the Total Maturation Score (TMS). Although assessments were conducted by two independent neuroradiologists with consensus procedures, the manual and lower-resolution nature of their methods suggests a moderate risk of bias due to limited reproducibility and greater susceptibility to measurement error.

**Table 3:** MRI data acquisition and analysis.

Study	Scanner	acquisition details	Post-processing	Risk of bias
Brembilla et al. (19)	1.5T with neonatal coil	T2: TR 6,000 ms/TE 200 ms, 3 mm slices, 0.3 mm gap; T1: TR 750 ms/TE 12 ms, same resolution; DWI acquired	Manual 2D area measurements of brain regions + Total Maturation Score (TMS) by two neuroradiologists	Moderate
Lodygensky et al. (17)	1.5T Marconi/Philips	T1 3D fast-GRE: TR 15 ms/TE 4.4 ms, 1.5 mm slices; T2 + PD FSE: TR 3,500 ms/TE 30,150 ms, in-plane 0.703 × 0.703 mm, no gap	k-NN tissue classification, VBM (SPM2), manual hippocampal segmentation using 3D Slicer	Low
Sacchi et al. (18)	3T Philips	T2 fast-spin echo: TR 14,730 ms/TE 160 ms, voxel size 0.86 × 0.86 × 2 mm, 1-mm overlap; T1 + DWI also acquired	PCA on 92 gray matter regions via Jacobian determinants; neonatal atlas used	Low

TR, repetition time; TE, echo time; T1, T1-weighted imaging; T2, T2-weighted imaging; DWI, diffusion-weighted imagine; PD, proton density; VBM, voxel-based morphometry; k-NN, k-nearest neighbors; SPM2, statistical parametric mapping version 2; GRE, gradient recalled echo; FSE, fast spin echo; FWHM, full width at half maximum; ROI, region of interest; TMS, total maturation score.

### 3.5 Study characterstics

Table 4 shows the study characteristics of the three articles. There was one retrospective case-controlled study, one retrospective observational study, and one secondary analysis of evaluation of preterm imaging study from a randomised control trial (RCT). Cohort year ranged from 2001 to 2018 with number of infants per study ranging from 26 to 314. Two studies used a 1.5 Tesla scanner, and one used a 3.0 Tesla scanner. All studies used Bayley Scale for Infant Development (BSID), one used Edition II and two used Edition III. However, one study used a composite score of BSID-II and Griffith's rating scale (19). In addition to BSID, Lodygensky et al. (17), used Assessment of Preterm Infant's behaviour (APIB), and Sacchi et al. (18), also used Modified-Checklist for Autism in Toddlers (M-CHAT).

**Table 4:** Study characteristics.

Study	Study type; participant number (FGR)	Cohort year	MRI	Inclusion criteria	Exclusion criteria	FGR definition	NDO scale and classification
Brembilla et al. (19)	Retrospective case-control study *n* = 84 (48)	2009–2018	1.5 Tesla scanner with a dedicated neonatal coil, including axial and coronal T2-weighted sequences, axial and sagittal T1-weighted sequences, and diffusion-weighted sequences	Infants born between 26- and 32-weeks’ gestation, undergoing a cerebral MRI at term equivalent age, admitted to the neonatal intensive care unit at Buzzi Children's Hospital	Twins, chromosomal anomalies, known fetal malformations, congenital infections, placental abruption, severe intrapartum events such as placental abruption, cord prolapse, and uterine rupture, serious motion artifacts, focal or diffuse lesions of the brain including intraventricular hemorrhage grade >1, periventricular leukomalacia (PVL, including non-cystic PVL), isolated punctate lesions, and cerebellar hemorrhages	Defined according to the international consensus definition based on ultrasound criteria, involving serial Doppler velocimetry ultrasound examinations of the umbilical artery (UA), middle cerebral artery (MCA), and ductus venosus (DV) until delivery	The Griffiths’ rating scale was used up to 2014, subsequently the Bayley Scale III was used. Composite scores of each scale were transformed into a development quotient, converting the raw score into an equivalent mental age using the formula: equivalent mental age/corrected age at the time of the test × 100
Lodygensky et al. (17)	Retrospective observational study *n* = 26 (13)	2001–2004	1.5 Tesla scanner (Marconi/Philips MR systems) was used, including a 3D fast-gradient echo sequence and a double echo (T2 and proton-density) fast-spin echo sequence	Infants born with IUGR secondary to placental insufficiency, defined by fetal measurements between 20 and 36 weeks of pregnancy and having abnormal Doppler flow values in the umbilical artery	Twins, chromosomal anomalies, known fetal malformations, congenital infections, placental abruption, severe intrapartum events (placental abruption, cord prolapse, uterine rupture), severe motion artifacts, and presence of focal or diffuse brain lesions diagnosed by ultrasound or MRI	Defined as a resistance to arterial umbilical flow higher than the 95th percentile measured by two indexes (resistance index [RI] and systolic/diastolic velocities [S/D])	Assessment of Preterm Infants’ Behavior (APIB) at term-equivalent age
							Bayley Scales of Infant and Toddler Development, Second Edition (Bayley II), assessing mental developmental index (MDI) and psychomotor developmental index at 24 months corrected age
Sacchi et al. (18)	Secondary analysis of evaluation of preterm imaging study data (e-Prime: a randomized control trial *n* = 314 (49)	2010–2013	Philips 3 Tesla (Philips Medical Systems) magnetic resonance system	Infants born before 33 weeks of gestation, mothers older than 16 years of age and not hospital inpatients	Major congenital malformation, metallic implants, parents unable to speak English, infant subject to child protection proceedings, BW < 10th percentile without antenatal growth adversity, major lesion on term MRI, cerebral palsy at follow-up assessment	Identified by reviewing medical discharge records, reported antenatal abnormalities on fetal scans and/or Doppler ultrasound velocimetry, clinical evaluation of IUGR, risk factors for IUGR combined with BW < 10th percentile for gestational age	Bayley Scales of Infant and Toddler Development, 3rd Edition; Modified-Checklist for Autism in Toddlers (M-CHAT)

FGR, fetal growth restriction; NDO, neurodevelopmental outcomes; *n*, number; MRI, magnetic resonance imaging.

### 3.6 Neurodevelopmental outcomes and MRI correlates for individual studies

The summary of findings are shown in Table 5. Brembilla et al. (19), reported no significant differences between FGR and AGA groups for gross motor, fine motor or cognitive scores (19). However, in the FGR cohort, those with absent or reverse end diastolic flow in umbilical artery as measured with doppler ultrasound had significantly worse gross motor scores than the FGR subgroup with typical end diastolic flow. While, in theory this might reflect subsequent adverse brain development, this was not borne out in MRI. Brembilla et al. (19), found no significant difference for MRI TMS or other single maturation parameter between FGR and AGA. The inner calvarium and parenchyma were significantly smaller in FGR subjects, as might be expected, although no significant differences were found for areas of the cerebellar hemispheres, the cerebellar vermis, and the lateral ventricles. When combining MRI data from all cerebral areas to neurodevelopmental outcomes, a statistically significant positive correlation between brain parenchyma area and cognitive scores was reported in FGR infants, but not AGA infants. Intriguingly, the mean cognitive quotient for FGR infants appeared to be higher (non-significant) than for AGA infants. As this was not significant the results are not an outlier. Multivariate analysis for demographic differences, which are significant between FGR and AGA, only appears to have been applied across all subjects, which confirms the positive correlation of parenchymal area and cognitive quotient. This means that potential confounders, such as gender, cannot be excluded from any cohort differences.

**Table 5:** Summary of neurodevelopmental outcomes (NDO) and MRI correlates in FGR infants.

Study	Assessment age	Motor		Cognitive		Behavioural	
		NDO	MRI correlate	NDO	MRI correlate	NDO	MRI correlate
Brembilla et al. (19)	24 months	No significant difference for gross and fine motor scores in FGR compared to AGA. However, in the FGR cohort, the subgroup with umbilical arterial doppler velocity alterations had significantly worse gross motor scores (*p* = 0.0005).	N/A	No significant difference in gross cognitive scores in FGR compared to AGA.	FGR group only—positive correlation between parenchyma areas and cognitive scores in FGR infants (*p* = 0.0097).	N/A	N/A
Lodygensky et al. (17)	24 months	N/A	N/A	No significant difference for mean MDI in FGR infants compared to AGA infants.	FGR group only—no correlations examined.	N/A	N/A
Sacchi et al. (18)	22 months	Very preterm FGR infants had lower motor scores compared to very preterm AGA infants (*p* = 0.018).	FGR group only—no correlations examined.	Very preterm FGR infants had lower cognitive scores compared to very preterm AGA infants (*p* = 0.018)	FGR group only—no correlations examined.	Very preterm infants had increased M-CHAT positive screening compared to AGA infants (*p* = 0.018)	FGR group only—no correlations examined.

FGR, fetal growth restriction; MRI, magnetic resonance imaging.

Lodygensky et al. (17), looked at the hippocampal volume in FGR infants, and the correlation of hippocampal volume on neurodevelopmental outcomes (17). Bayley II assessments were undertaken, but only the mental developmental index (MDI), and not the psychomotor developmental index was reported. No statistical difference between FGR infants and AGA infants was found. Further, 35% of the cohort were lost to follow-up, and it is not reported whether the attrition was equal between FGR and AGA groups. Hippocampal volume correlated with MDI for the entire cohort, but there was no intergroup comparison of MDI and hippocampal volume, meaning the importance of hippocampal volume to FGR outcomes could not be established.

Sacchi et al. (18), showed very preterm FGR infants had significantly lower motor scores and significantly lower cognitive scores compared to very preterm AGA infants at 22 months of age (18). They also demonstrated very preterm FGR infants had increased M-CHAT positive screening compared to AGA infants. On MRI, there were significant adjusted smaller volumes in the limbic region, and larger volume differences in the fronto-insular, temporo-parietal and frontal regions of FGR infants. Across all subjects, cognitive scores were associated with larger frontal and occipital volumes, and lower motor scores were associated with larger parietal volumes. However, there was no intergroup comparison, and so no capacity to identify which MRI marker might be key for FGR outcomes.

## 4 Discussion

In this systematic review of the literature on MRI to predict adverse neurodevelopmental outcomes in FGR infants, three studies met the inclusion criteria. Although all the studies showed a correlation between certain regions of the brain and neurodevelopmental outcomes across all studied infants, these results were not separated into FGR and AGA cohorts for comparison. This resulted in limited FGR-specific insights and meant that the question of the impact of FGR on an infant could not be addressed in those studies. There was also no data available in these papers for extraction to undertake a meta-analysis.

Recent work has quantified several structural and metabolic differences between AGA and FGR brains. Reduced total brain and cerebellar volumes and lower ADC values were identified in multiple white matter and grey matter regions in FGR fetuses (22). Nonetheless, efforts to detect areas of meaningful early cerebral change with accompanying later neurodevelopmental deficits in FGR infants have produced few and varied results. Two studies did examine correlations between neurodevelopmental outcomes and MRI results in an FGR cohort (19, 23). One of these studies was excluded from the analysis because the MRI was undertaken at 12 months (23). Padilla et al. (23), found reduced grey matter volume in the left precuneus and right superior-frontal gyrus that correlated with reduced motor scores in preterm FGR infants. While reduced grey matter in the right superior parietal gyrus correlated with reduced adaptive behaviour subscale scores in preterm FGR infants (23). For our purposes in this study, an MRI undertaken at 12 months of corrected age, as in Padilla et al. (23), lies outside the parameters of neonatal MRI prognostication. This component of our question is vital because a child that would otherwise be at risk of poor neurodevelopmental outcome could potentially receive therapeutic interventions prior to the age of 12 months, which could have a profound impact on their development.

Brembilla et al. (19), described a positive correlation between the entire parenchymal area and cognitive scores in FGR infants (19). This was a result of combining MRI data from all cerebral areas to neurodevelopmental outcomes. However, between these two studies there were differing MRI time points, differing MRI parameters and brain regions examined. Brembilla et al. (19), used a 1.5 Tesla MRI scanner at term equivalent corrected age and used TMS parameters to examine myelination, cortical folding, glial cell migration pattern and germinal matrix distribution. In addition, they manually measured cerebral areas such as the inner calvarium, cerebral parenchyma, cerebral hemispheres, and cerebellar vermis. Padilla et al. (23), used a 3.0 Tesla MRI scanner at 12 months corrected age and analysis of multiple brain regions was made by automated lobar volumetry and VBM. As a result of the very different techniques used between studies, it is difficult to draw conclusions.

There are several studies including some FGR infants that examined brain MRI and neurodevelopmental outcomes but did not meet the selection criteria (24, 25). Ball et al. (24), reported on preterm infants born before 33 weeks gestation, with MRI available at term-equivalent age in 449, of whom 425 had a 2-year neurodevelopmental assessment with Bayley Scales of Infant and Toddler Development, Third Edition (BSITD-III); 14.7% of the cohort were small for gestational age. The aim was to use data-driven, multivariate methods to test the hypothesis that brain development is altered by multiple environmental factors interacting with early extrauterine exposure following preterm birth. One factor in a cluster of “Intrauterine Compromise and Growth Restriction” was FGR, and collectively this group had a global decrease in brain volume, alongside increased T2 signal intensity suggestive of increased CSF volume apparent in the fourth ventricle and surrounding the brainstem and cerebellum, decreased T2 signal intensity in the lateral ventricles, and localized increases in FA in the corpus callosum. However, it was not possible to extract the preterm FGR cohort from the manuscript and hence it did not meet criteria for inclusion.

Barnett et al. (25), examined a cohort of 491 infants without focal destructive brain lesions born before 34 weeks, who underwent term equivalent structural and dMRI with 381 infants having BSITD-III assessment at 20 months. 17.3% of the cohort had FGR as defined by the obstetric team but criteria were not given. dMRI data were analyzed using tract based spatial statistics and the relationship between dMRI measures in white matter and individual perinatal risk factors assessed to test the hypothesis that increased exposure to perinatal risk factors was associated with lower fractional anisotropy (FA), and higher radial, axial and mean diffusivity (RD, AD, MD) in white matter. Neurodevelopmental performance was investigated to test the hypothesis that lower FA and higher RD, AD and MD in white matter were associated with poorer neurodevelopmental performance. FGR was identified as one of a number of factors associated with diffuse white matter injury and lower FA values, and lower FA values in turn were associated with subsequent lower neurodevelopmental performance. However, it was not possible to extract the FGR group data and their outcomes, and hence the study did not meet criteria for inclusion. There were a number of strengths to this study. A literature search was undertaken in four databases with screening and quality assessment performed by two independent investigators with a third to resolve disputes. However, the quality of included studies ranged from moderate/high to low/moderate. All studies used standardised neurodevelopmental tests such as BSID.

Multiple limitations can be identified. Primarily, most studies were insufficiently powered to allow FGR and AGA comparative analysis, and most failed to report values for their outcomes, instead employing correlation plots.

Different brain regions were examined in each of the studies. Lodygensky et al. (17), examined only the hippocampus, a region that has been shown to be vulnerable to injury in the FGR neonate (7). Padilla et al. (23), did not observe differences in hippocampal volume in their study. However, this discrepancy may be due to the differing time points of MRI examination: at term equivalent age vs. 12 months corrected age. A more consistent approach to MRI analysis is yet to be used in studies such as these.

In addition, these three studies spanned a long timeframe (2008–2021) which introduces variability due to advances in MRI technology and development tools (BSID version). Variable results for neurodevelopmental outcomes were reported. Two of the three studies did not find any significant adverse neurodevelopmental outcomes at assessment (17, 19). This may have been due to the exclusion criteria for Brembilla et al. (19), and Lodygensky et al. (17), as both studies excluded infants with minor MRI findings of focal or diffuse brain lesions. Hence the cohort of FGR infants in these studies may have had overall better expected neurodevelopmental outcomes. Furthermore, examining early MRI markers with cognitive and motor outcomes at a later time point might highlight additional correlations; such as at school age when many of these adverse neurodevelopmental outcomes are commonly observed in FGR children (3, 4). Assessment age of 24 months is too low to accurately detect the possible onset of other neurodevelopmental disorders such as autism spectrum disorder, attention deficit hyperactivity disorder, and intellectual disability. A longer follow-up would be beneficial to detect these disorders and study the potential ability to detect them early using MRI. This is the reason why our criteria for outcome data was greater than 12 months of age. However, it should be noted that many MRI methods are predictive of toddler/childhood outcomes, and several studies provide clinical evaluations which are also predictive or correlative of outcome at term equivalent age in FGR cohorts (12, 26). The other salient feature of this review was the finding that all included studies used a preterm cohort and therefore lacked the inclusion of term FGR infants. This is despite a search criterion open to term births. While preterms are an important cohort, this means we could not examine whether any observed changes were secondary to prematurity or FGR. Therefore, there is a vast gap in knowledge of FGR MRI and neurodevelopmental outcomes in the large cohort of term and near term born FGR babies.

From the review of the literature and subsequent learnings from deficiencies in current published studies we offer further recommendations which may be helpful for the design of potential future studies. Other than including preterm and term FGR cohorts, as well as AGA cohorts, the classification of FGR should be consistent. This could mean that the presence or absence of placental insufficiency is included as part of trial inclusion criteria. The infants should also be classified on whether brain lesions are present or absent. This would be important in the classification of high risk of disability or not. Attention to details of acquisition, and pre- and post-processing methods are important to adequate image quality and resolution and should be included in the manuscripts (27). Further information to inform pertinent questions for healthcare institutions and clinicians should also be gathered such as is universal MRI screening in FGR infants warranted, is it economically justified, does it have an impact on parental/family experience, and does it have meaningful impact on outcome. Early diagnosis is crucial to mitigate poor neurodevelopmental outcomes in newborns at risk. FGR is such a risk, and MRI has the potential to stratify that risk. Unfortunately, current evidence is insufficient to firmly establish MRI prognostic capabilities specifically for FGR infants. It was not possible to conduct a meta-analysis, yet a non-systematic review could provide interesting insights and suggestions for future research. This study showed gaps in the literature which need further addressing. The study design approaches used to date do not facilitate sub-group analysis and specific regions of interest that could be utilised as future biomarkers in FGR infants would be valuable. Neonatal MRI measures that correlate with or predict childhood outcome need specific validation or sub-group analysis for FGR. This vulnerable population would benefit from this technique to detect possible abnormalities early and provide treatment. Further research that is sufficiently powered to analyse an FGR cohort, along with the use of advanced MR acquisition and analytic techniques is needed before a robust clinical marker of adverse brain development in the FGR newborn is likely to be identified, prospectively tested and then used in clinical practice.

## Data Availability

The original contributions presented in the study are included in the article/Supplementary Material, further inquiries can be directed to the corresponding author.
