# Supplementary material for: Neonatal brain MRI to prognosticate neurodevelopmental outcomes in fetal growth restricted infants: a systematic review
Source: Front Pediatr. 2026 Jan 12;13:1681205. doi: 10.3389/fped.2025.1681205 (PMC12832895; doi:10.3389/fped.2025.1681205)
Supplement: Supplementary file 1 [file Table1.docx]

| Not relating to SGA or FGR population | |
| --- | --- |
| *Article* | *Rational* |
| Barnett et al. (2018) 113 | Preterm: infants born at <34 weeks with no FGR neurodevelopmental outcomes |
| Choi et al. (2021) 254 | Inclusion: infants with a birth weight of <1500g. Preterm group (n=60) of which n=10 is SGA. The SGA population is not separated apart from the larger preterm group. |
| George et al. (2021) 444 | No comparison population. Also, they identify 15 SGA (<10^th^ percentile birthweight) in the “Early” group, but their individual data is not present. |
| Kline et al (2020) 674 | Weight not considered. Inclusion criteria were ≤31 weeks. |
| Lee et al. (2020)  712 | Very preterm infants with birth weight of <1.5 kg. No mention of percentiles. |
| Lind et al. (2009) 740 | VLBW: Inclusion: <1501g & <37 weeks’ gestation. SGA embedded within VLBW group. |
| Maeda et al. (2019) 773 | “Fetal growth restriction, defined as birth weight below the 10th percentile of the Japanese standard [16], was present in 36% of the subjects” However, the data is not segmented into an SGA/FGR (Japanese standard) group |
| Munck et al. (2009) 876 | Exclusion criteria: “The exclusion criteria were congenital anomalies for syndromes, mother’s self-reported use of illicit drugs or alcohol during pregnancy, and birth weight<2.0 SD (small for gestational age, SGA)” |
| Parikh et al. (2013) 956 | Incorrect population: ELBW, <1000g |
| Pogribna et al. (2014) 1002 | ELBW used (≤1000g); from this group (n=39), 18% were SGA (<10^th^ percentile). However, there is not separate findings/outcomes for this subset so it would not be possible to draw conclusions from this study. |
| Vegni et al. (1994) 1296 | No indication of SGA, MRI. Birth weights as categories <1000g, 1001-1250g; CT, EEG, and NMR, no MRI |
| Woodward et al. (2006) 1335 | Not explicitly stated, but SGA is included in their analyses. “Small size for gestational age was defined as a birth weight more than 2 SD below the mean for gestational age and sex.” Findings for SGA are in table; therefore, data can be used. |
| Young et al (2016) 1370 | IUGR matches definition; however, not split into groups (i.e., AGA vs. SGA). |
| Not primary peer reviewed | |
| *Article* | *Rational* |
| Andreato et al. (2021) 70 | Abstract only |
| Renzo et al. (2021) 173 | Abstract only |
| Guidotti et al. (2012) 492 | Abstract only |
| Okuma et al. (2011) 920 | Abstract only |
| Rose et al. (2012) 1086 | Abstract and poster only |
| Suffren et al. (2016) 1214 | Abstract and poster only |
| No MRI imaging modality | |
| *Article* | *Rational* |
| deBie et al. (2015) 305 | MRI at 4-7 years. Novelty of this study was fMRI in young SGA children |
| Halevy et al. (2021) 498 | Foetal brain MRI |
| MRI at 12 months | |
| *Article* | *Rational* |
| Batalle et al. (2012) 124 | MRI at 12­ ± 2 months CA |
| Padilla et al. (2011)  941 | MRI at 12 ± 2 months CA |
| Not in English | |
| *Article* | *Rational* |
| Imamura et al. (1996 572 | Not in English (abstract in English, article in Japanese) |
